# Targeting Hippo coactivator YAP1 through BET bromodomain inhibition in esophageal adenocarcinoma

**DOI:** 10.1002/1878-0261.12667

**Published:** 2020-04-07

**Authors:** Shumei Song, Yuan Li, Yan Xu, Lang Ma, Melissa Pool Pizzi, Jiankang Jin, Ailing W Scott, Longfei Huo, Ying Wang, Jeffrey H. Lee, Manoop S. Bhutani, Brian Weston, Namita D Shanbhag, Randy L. Johnson, Jaffer A. Ajani

**Affiliations:** ^1^ Department of Gastrointestinal Medical Oncology The University of Texas MD Anderson Cancer Center Houston TX USA; ^2^ Department of Gastroenterology, Hepatology & Nutrition The University of Texas MD Anderson Cancer Center Houston TX USA; ^3^ Department of Cancer Biology The University of Texas MD Anderson Cancer Center Houston TX USA

**Keywords:** BRD4, CSCs, esophageal cancer, Hippo/YAP1, JQ1, therapy resistance

## Abstract

Hippo/YAP1 signaling is a major regulator of organ size, cancer stemness, and aggressive phenotype. Thus, targeting YAP1 may provide a novel therapeutic strategy for tumors with high YAP1 expression in esophageal cancer (EC). Chromatin immunoprecipitation (ChiP) and quantitative ChiP‐PCR were used to determine the regulation of the chromatin remodeling protein bromodomain‐containing protein 4 (BRD4) on YAP1. The role of the bromodomain and extraterminal motif **(**BET) inhibitor JQ1, an established BRD4 inhibitor, on inhibition of YAP1 in EC cells was dissected using western blot, immunofluorescence, qPCR, and transient transfection. The antitumor activities of BET inhibitor were further examined by variety of functional assays, cell proliferation (MTS), tumorsphere, and ALDH1+ labeling *in vitro* and *in vivo.* Here, we show that BRD4 regulates YAP1 expression and transcription. ChiP assays revealed that BRD4 directly occupies YAP1 promoter and that JQ1 robustly blocks BRD4 binding to the YAP1 promoter. Consequently, JQ1 strongly suppresses constitutive or induced YAP1 expression and transcription in EC cells and YAP1/Tead downstream transcriptional activity. Intriguingly, radiation‐resistant cells that acquire strong cancer stem cell traits and an aggressive phenotype can be effectively suppressed by JQ1 as assessed by cell proliferation, tumorsphere formation, and reduction in the ALDH1+ cells. Moreover, effects of JQ1 are synergistically amplified by the addition of docetaxel *in vitro* and *in vivo*. Our results demonstrate that BRD4 is a critical regulator of Hippo/YAP1 signaling and that BRD4 inhibitor JQ1 represents a new class of inhibitor of Hippo/YAP1 signaling, primarily targeting YAP1 high and therapy‐resistant cancer cells enriched with cancer stem cell properties.

## Graphical abstract

This study, for the first time, demonstrates that BRD4 is a critical regulator of YAP1 through direct occupancy on its promoter, while JQ1 decreases YAP1 expression and its transcription output (CTGF, SOX9, and Cyr61), suppresses CSC traits, and decreases ALDH1+ cells, indicating targeting YAP1 can be achieved through BET inhibition, which provides a new therapeutic strategy for EC.

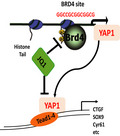

**Abbreviations**
BET: the bromodomain and extraterminal protein
ChiP: chromatin immunoprecipitation
CSCs: cancer stem cells
EAC: esophageal adenocarcinoma
EC: esophageal cancer
ESCA: esophageal carcinoma
ESCC: esophageal squamous cell carcinoma
qPCR: quantitative real‐time PCR
SOX9: transcription factor SOX‐9
TCGA: The Cancer Genome Atlas
VP: verteporfin
YAP1: yes‐associated protein 1

## 1 Introduction

Esophageal cancer (EC) is highly virulent and a major global health burden with more than 600 000 new cases each year (Torre *et al.*, 2016). Esophageal adenocarcinoma (EAC), the predominant histology in the United States, has a rising incidence in the West (Brown *et al.*, 2008; Torre *et al.*, 2016). EAC is often diagnosed late based on symptoms, and cure rates are only 15% for all comers (Ajani *et al.*, 2015). The progress against EAC has been painfully slow, while some of the critical genetic alterations have been discovered over the years and recently in a more comprehensive manner (Cancer Genome Atlas Research *et al.*, 2017). Much more investigations are needed to translate into clinical therapeutics. Thus, novel treatment strategies that are based on molecular rationale are highly desirable.

The Hippo/YAP1 plays a critical role in modulating organ size, stem cell maintenance, and cell proliferation (Tumaneng *et al.*, 2012a; Tumaneng *et al.*, 2012b). High YAP1 nuclear expression correlates with poor patient outcome in EAC and some other cancers (Kang *et al.*, 2011; Xu *et al.*, 2009). Overexpression YAP1 in tumor cell lines can induce EMT and enhances *in vitro* invasion (Overholtzer *et al.*, 2006). Tissue‐specific expression of YAP1 in the liver in genetic mice results in liver overgrowth and tumor formation (Camargo *et al.*, 2007). In addition, YAP1 is highly related to constitutive and target therapy resistances (Flaherty *et al.*, 2015; Lin *et al.*, 2015; Zeng and Hong, 2008) and YAP1 has been reported often as a terminal node of many oncogenic pathways (Keren‐Paz *et al.*, 2015; Lin *et al.*, 2015). Furthermore, the 11q13 locus containing YAP1 has been reported to be amplified in EAC (Cancer Genome Atlas Research *et al.*, 2017; Lockwood *et al.*, 2012). As its oncogenic function relies on its nuclear localization, how to effectively target nuclear YAP1 is still a challenge for clinical utility.

The activity of nuclear YAP1 as coactivator is controlled by the canonical Hippo signaling through its upstream kinase cascades (Yu and Guan, 2013; Yu *et al.*, 2015). The Hippo/YAP1 pathway comprised of a core kinase cascade in that Mst1/2 kinase phosphorylates LATS1/2 kinase and then LATS1/2 phosphorylates and represses the transcriptional coactivators YAP1 and TAZ by promoting YAP1 ubiquitination and degradation (Yu and Guan, 2013). In cancers, aberrant activation of YAP1 has been reported by divergent signaling and pathways such as mechanical forces, PI3K, and G protein‐coupled receptor signaling (Yu *et al.*, 2014; Yu *et al.*, 2012a; Yu *et al.*, 2012b).

Inappropriate activation of YAP1 may also be controlled by epigenetic regulators. The BET family include BRD2, BRD3, BRD4, and BRDT, which share common domain architecture (Dhalluin *et al.*, 1999; Filippakopoulos *et al.*, 2010). BET proteins bind to acetylated lysine residues in histones, recruit chromatin‐modifying enzymes to target genes’ promoter, and function as coregulators in a context‐dependent manner (Dhalluin *et al.*, 1999; Filippakopoulos *et al.*, 2010). BRD4 is the most cancer‐related BET family member, and studies have revealed important roles for BRD4 protein in certain types of cancer (Shi *et al.*, 2015; Shu and Polyak, 2016). Overexpression of BRD4 was found in melanoma, and downregulation of BRD4 by genetic knockdown is sufficient to recapitulate the antitumoral effect of BET inhibitors in melanoma cell (Segura *et al.*, 2013). Zuber *et al.* (2011) showed that inhibition of BRD4 using shRNAs or the small‐molecule inhibitor JQ1 led to dramatic antileukemic effects *in vitro* and *in vivo*. BRD4 is reported to be upregulated in other oncogenic signaling and cancer stem cell (CSC) signaling such as Hh pathways Gli‐1 (Tang *et al.*, 2014) and Jagged1/Notch1 (Andrieu *et al.*, 2016). Inhibition of BRD4 by BET inhibitors suppresses ALDH1 activity by targeting ALDH1A1 superenhancer in ovarian cancer (Yokoyama *et al.*, 2016). Targeting BET proteins improve the therapeutic efficacy of BCL‐2 inhibition and PARP inhibition in leukemia, breast cancer, and ovary cancer, respectively (Peirs *et al.*, 2017; Yang *et al.*, 2017). BRD4 controls the transcription of their target genes either directly or indirectly, and specific BET inhibitors are in several clinical trials (NCT02543879, NCT02158858, etc.). We proposed that BRD4 epigenetically regulated YAP1 expression and transcription and targeted aberrant activation of YAP1 in EAC could be reduced by BET inhibition.

In this study, we provide evidence that BRD4 is an important regulator of YAP1 transcription through direct occupancy of its promoter that is markedly inhibited by the BET inhibitor JQ1. JQ1 effectively decreased YAP1 expression and its transcription activity in the constitutively high or induced YAP1 EAC cells. Remarkably, JQ1 can effectively suppress therapy‐resistant EAC cells endowed with CSC traits and decreases ALDH1+ cells (representing CSCs). Furthermore, the effect of JQ1 is amplified when it is combined with docetaxel *in vitro* and *in vivo*.

## 2 Materials and methods

### 2.1 Cells and reagents

We used the human EAC cell lines BE3, Flo‐1, SKGT‐4, JHESO, and OACP, and radiation‐resistant EAC cell line Flo‐1 XTR that have been described previously (Raju *et al.*, 2006; Soldes *et al.*, 1999; Wang *et al.*, 2012). All cell lines were authenticated at the Characterized Cell Line Core at UT MD Anderson Cancer Center regularly. JQ1 was obtained from Selleck Chemicals (St. Louis, MO, USA). Doxycycline (Dox) was from Sigma‐Aldrich. Anti‐YAP1 purchased from Cell Signaling Technology was previously described. Anti‐SOX9 and BRD4 antibodies were obtained from EMD Millipore (Massachusetts 01821). Antibodies against β‐catenin, EGFR, phosphor‐S6, phosphor‐70S6K, and c‐MYC were from Cell Signaling Technology (Boston, MA 02241‐3843). BRD4 plasmid (pcDNA2‐BRD4) and doxycycline‐inducible YAP1 lentiviral plasmid (PIN20YAP1) were previously reported (Song *et al.*, 2017).

### 2.2 Cell survival assay

The EAC cells and their resistant clones were treated with 0.1% dimethyl sulfoxide (control) and JQ1 at various concentrations of 0.25 μm, 0.5 μm, 1 μm, 2 μm, 4 μm, 8 μm, and 16 μm for 3 days and 6 days. For combination treatment experiments, JQ1, docetaxel, or a combination at various concentrations was administered for 3 days and 6 days and the cell viability was assessed using an MTS assay as described previously (Song *et al.*, 2013). GraphPad Prism7 was used for IC50 analysis by following the instruction from GraphPad.com (https://www.graphpad.com/support/faq/how-to-determine-an-icsub50sub/). All assays were in triplicate.

### 2.3 Western blot analysis

Proteins were isolated from EAC cells treated with JQ1 and analyzed using western blotting as described previously (Song *et al.*, 2009).

### 2.4 Luciferase reporter assays

YAP1/Tead1 transcriptional activity was determined using the 5×‐UAS‐luciferase reporter and Gal4‐TEAD4 constructs as described previously (Zhao *et al.*, 2009). Transient cotransfection of EAC cells using 5×‐UAS‐luciferase reporter and Gal4‐TEAD4 with a CMV‐β‐gal construct with a Renilla vector was performed as described previously (Song *et al.*, 2013).

### 2.5 Tumorsphere assay

Tumorsphere assay was performed as described previously (Song *et al.*, 2013). Briefly, a single‐cell suspension of Flo‐1 cells and radiation‐resistant Flo‐1 XTR cells were seeded in triplicate onto a 24‐well ultralow attachment plate (800 cells/well) at designated medium as described previously (Song *et al.*, 2013). After 10–20 days of culture, the tumorspheres that formed (diameter > 100 μm) were counted through a microscope.

### 2.6 Immunohistochemistry

Immunohistochemistry (IHC) staining for YAP1, SOX9, and Ki67 was performed on xenograft tumor tissues of SKGT‐4 DOX+ and JHESO xenograft tumors using antibodies against SOX9 (1 : 2000) and YAP1 (1 : 100) and KI67 (1 : 100) as described previously (Song *et al.*, 2014; Song *et al.*, 2013).

### 2.7 Indirect immunofluorescence

Indirect immunofluorescent staining for nuclear expression of YAP1 and SOX9 in EAC cells was performed as described previously (Song *et al.*, 2009).

### 2.8 Real‐time polymerase chain reaction

To determine the alteration of YAP1 and its targets in response to BRD4 upregulation, quantitative real‐time PCR was performed using Applied Biosystems QuantStudio 3 System (Applied Biosystems, Foster City, CA, USA) using primers (Fig. S1) for BRD4, YAP1 and YAP1 targets, CTGF, SOX9, and Cy61 to detect their mRNA levels. The 7900 sequence detection system 2.2 software (Applied Biosystems) automatically determined the fold change for these genes in each sample by using the δδCt method with 95% confidence.

**Fig. 1:**
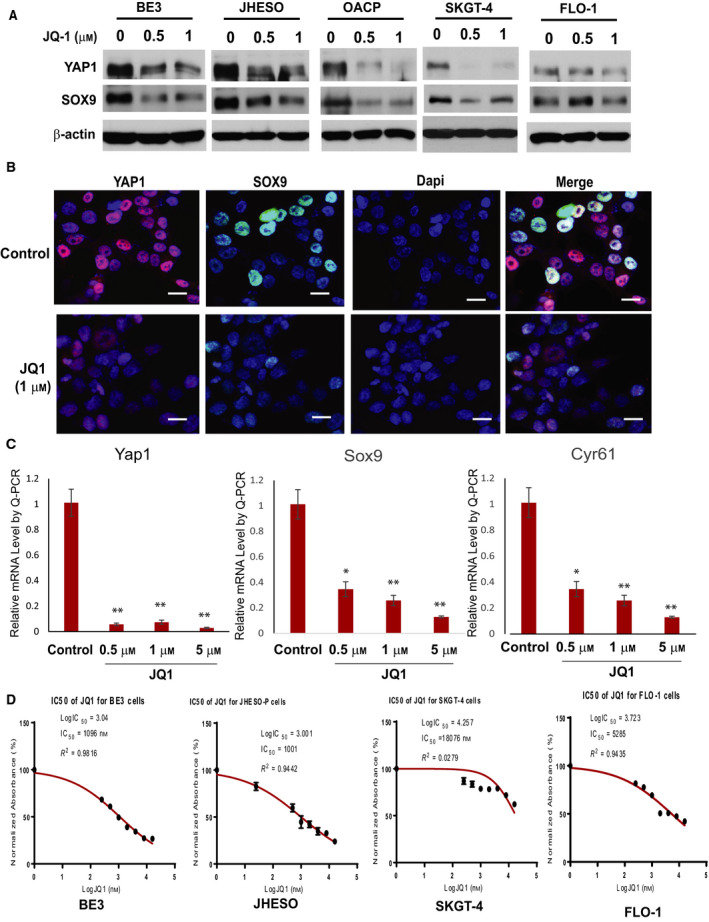
The JQ1 suppresses YAP1 transcriptional output and its expression in EAC cells. (A) Cell lysate from BE3, FLO‐1, JHESO, OACP and SKGT‐4 EAC cells treated with JQ1 at 0.5 1 μm and 1 μm for 48 h was performed with western blotting for YAP1 and SOX9 using their specific antibodies as described in Materials and Methods. (B) Immunofluorescent staining (IF) for SOX9 and YAP1 in JHESO cells treated with or without JQ1 at 1 μm was performed as described in Materials and Methods and observed under confocal microscopy. Scale bar, 25 μm; (C) mRNA levels of YAP1 and its target SOX9 and Cyr61 were detected by qPCR in JHESO EAC cells upon treatment with JQ1 at 0.5 μm, 1 μm, and 5 μm for 48 h as described in Materials & Methods. Values shown represent the mean and SD of at least triplicate assays for all experiments. *P* value was shown each treatment group compared with control (*P < 0.05; **P < 0.01). (D) The EAC cells were treated with the doses indicated of JQ1 for 6 days, and then, CellTiter 96 AQueous One Solution was added to each well followed by 2 h of incubation at 37 °C and absorbance reading at OD 490. GraphPad Prism7 was used for IC50 analysis as described in Materials & Methods

### 2.9 *In vivo* xenograft mouse model

SKGT‐4 (PIN20YAP1) cells (1 × 10^6^) without (Dox−) or with (Dox+) YAP1 induction by doxycycline were inoculated into nude mice (*n* = 5/group) and fed with drinking water as described previously (Song *et al.*, 2017). After 10 days, JQ1 was intraperitoneally injected into mice in the Dox+ group at 30 mg·kg^−1^ per mouse three times a week for total 3 weeks.

In the JHESO CDX model, 2 × 10^6^ JHESO cells were subcutaneously injected into nude mice (*n* = 5/group). After about 10 days, when tumor reached around 50 mm^3^, the tumor‐bearing mice were equally divided four groups and underwent treatment as follows: control, JQ1 alone (intraperitoneal injection of 30 mg·kg^−1^ per mouse), docetaxel at 1 mg·kg^−1^ per mouse, or a combination of them three times a week for total 3 weeks. The mice’s tumor volumes, tumor weights, and body weights were measured as described previously (Song *et al.*, 2014; Song *et al.*, 2015). All measurements were compared using an unpaired Student *t*‐test.

### 2.10 Statistical analysis

Data were analyzed using the Student *t*‐test and Fisher exact test (for immunohistochemistry) as described previously (Song *et al.*, 2017). We used a 2‐sided significance level of *P* < 0.05 for all statistical analysis. Correlation between expression level and clinical pathologic data was analyzed by chi‐square or Kendall tau‐b (K) analysis using SPSS 22.0 (IBM, Armonk, NY, USA).

## 3 Results

### 3.1 JQ1 effectively inhibits YAP1 expression and transcription in EAC cells

We previously reported that YAP1 plays an important role in maintaining EC cell growth, therapy resistance, and endowment of CSC properties (Song *et al.*, 2014; Song *et al.*, 2015). Targeting YAP1 is a novel strategy in EC. To identify a clinical useful YAP1 inhibitor, we took advantage of BET bromodomain inhibitors (e.g., JQ1) that has been in clinical trials for many tumor types and proven safe. To determine whether JQ1 suppresses YAP1 expression and transcription, several EAC cell lines SKGT‐4, BE3, Flo‐1, JHESO, and OACP were treated with JQ1 at relative low concentration as indicated for 48 h, we found that JQ1 suppressed YAP1 and its target SOX9 expression in EAC cell lines (Fig. 1A). Immunofluorescent staining further confirmed the nuclear expression of YAP1 and SOX9 was dramatically reduced upon treatment with JQ1 at 1 μm for 24 h in JHESO and SKGT‐4 cells (Fig. 1B). Similarly, transcription of YAP1 and its target genes SOX9 and Cyr61 was significantly reduced in a dose‐dependent manner (Fig. 1C). This indicates that JQ1 is a strong inhibitor for YAP1 and its targets in EAC cells.

To further determine whether JQ1 affects EAC cell proliferation activity, five EAC cell lines were treated at various concentrations (0, 0.25 μm, 0.5 μm, 1 μm, 2 μm, 4 μm, 8 μm, and 16 μm) for 3 days and 6 days. As shown in Figs 1D and S1, JQ1 significantly inhibited EAC cell proliferation in a dose‐dependent manner in all five EAC cells with varied antiproliferative activities among these EAC cell lines.

### 3.2 BRD4 positively regulates YAP1 and its downstream targets

**Fig. 2:**
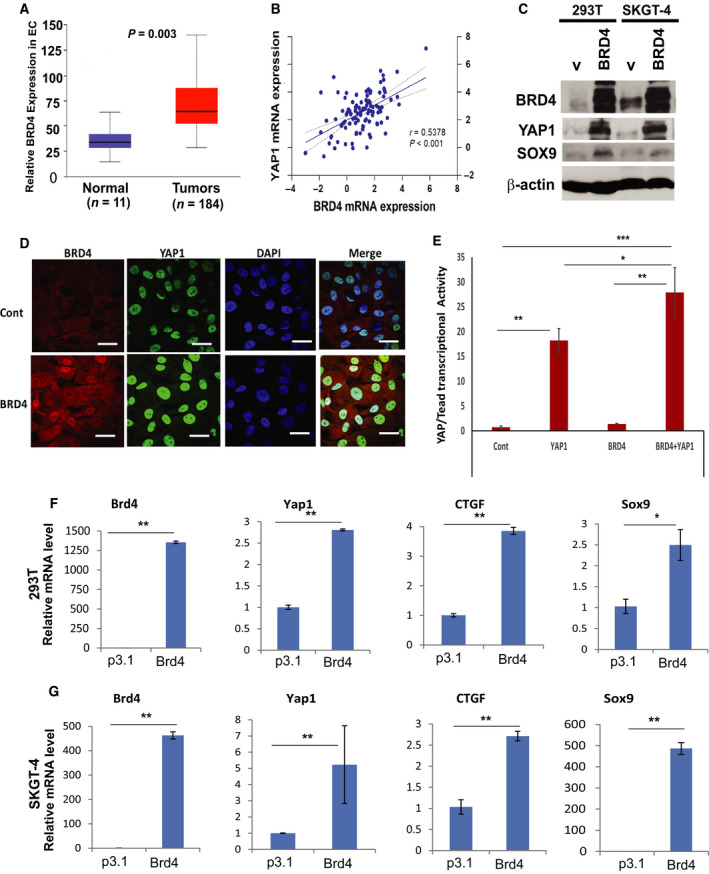
Bromodomain‐containing protein 4 positively regulates YAP1 expression and its downstream targets. (A) Expression of BRD4 in tumor tissues of 184 EC patients compared with normal control from TCGA database. (B) BRD4 and YAP1 mRNA levels were detected using quantitative PCR on 104 cases of EACs. Pearson’s test was used to evaluate the association between BRD4 and YAP1 mRNA level. Positive correlation was found between BRD4 and YAP1, *r* = 0.5378 (*P* < 0.001). (C) Cell total protein lysate from 293T and SKGT4 EAC cells with or without transfection of BRD4 cDNA (pcDNA2‐BRD4) was performed for western blotting for YAP1, BRD4, and SOX9. (D) IF staining of BRD4 and YAP1 in SKGT4 cells with or without transfection of BRD4 cDNA (pcDNA2‐BRD4) was observed by confocal microscopy. Scale bar, 25 μm; (E) Transcriptional activity of YAP1/Tead was detected by cotransfection of Gal4‐Tead and 5XUAS‐luciferase, YAP1 cDNA with or without BRD4 cDNA into SKGT4 cells. (F and G) mRNA levels of BRD4, YAP1, and its target CTGF and SOX9 were quantified by qPCR in both 293T (F) and SKGT4 EAC cells (G) with or without transfection of BRD4 cDNA (pcDNA2‐BRD4) as described in Materials & Methods. Values shown represent the mean and SD of at least triplicate assays for all experiments. *P < 0.05; **P < 0.01

To elucidate the molecular mechanisms by which JQ1 suppresses YAP1 expression and transcription, we focused on BRD4, one of BET family members of chromatin reader protein that are highly upregulated in EC tissues (184 cases) compared with normal tissues (11 cases) from TCGA database (Fig. 2A). We further assessed the association between BRD4 expression and tumor size from our 87 cases of EACs and found that BRD4 mRNA levels detected by qPCR were significantly associated with tumor size (Fig. S2A), poor tumor differentiation (Fig. S2B), and shorter survival (Fig. S2C). BRD4 increased the transcription of key genes involved in embryonic stem cell maintenance and oncogenes by either regulating chromosome remodeling or directly binding to enhancers or promoters of target genes (Alghamdi *et al.*, 2016; Gilan *et al.*, 2016; Yokoyama *et al.*, 2016). We hypothesized that BRD4 is a transcriptional cofactor for YAP1. Then, we analyzed BRD4 and YAP1 mRNA level using qPCR in 104 cases of EACs and evaluated the correlation between BRD4 and YAP1 expressions. As shown in Fig. 2B, we found that the expression of BRD4 was significantly correlated with the expression of YAP1 (*r* = 0.5378; *P* < 0.001). To further validate the positive association between these two, we transfected BRD4 cDNA into HET293T and SKGT4 EAC cell lines as indicated in Fig. 2C. YAP1 expression was dramatically increased in both cell lines, so does its target SOX9 expression in concert with the increased expression of BRD4. Increased YAP1 nuclear localization was seen in BRD4‐transfected cells compared with control cells (Fig. 2D). Further, YAP/TEAD luciferase activity was synergistically increased by cotransfection of both BRD4 and YAP1 with Gal4‐Tead and 5XUAS‐luciferase plasmids (Zhao *et al.*, 2009) in EAC cells (Fig 2E). To further determine whether BRD4 affects transcription of YAP1 and its transcriptional output, we detected YAP1 and its several target genes in BRD4‐transfected HET293T and SKGT4 cells. We found that increased BRD4 by transfection of BRD4 cDNA into these cells significantly upregulated mRNA levels of YAP1 and its targets SOX9 and CTGF (Figs 2F,G and S3). These data suggested that BRD4 strongly activated YAP1 and its transcriptional output in multiple EAC cell lines.

### 3.3 BRD4 regulates YAP1 transcription through binding its promoter and JQ1 suppresses BRD4‐mediated YAP1 transcription

**Fig. 3:**
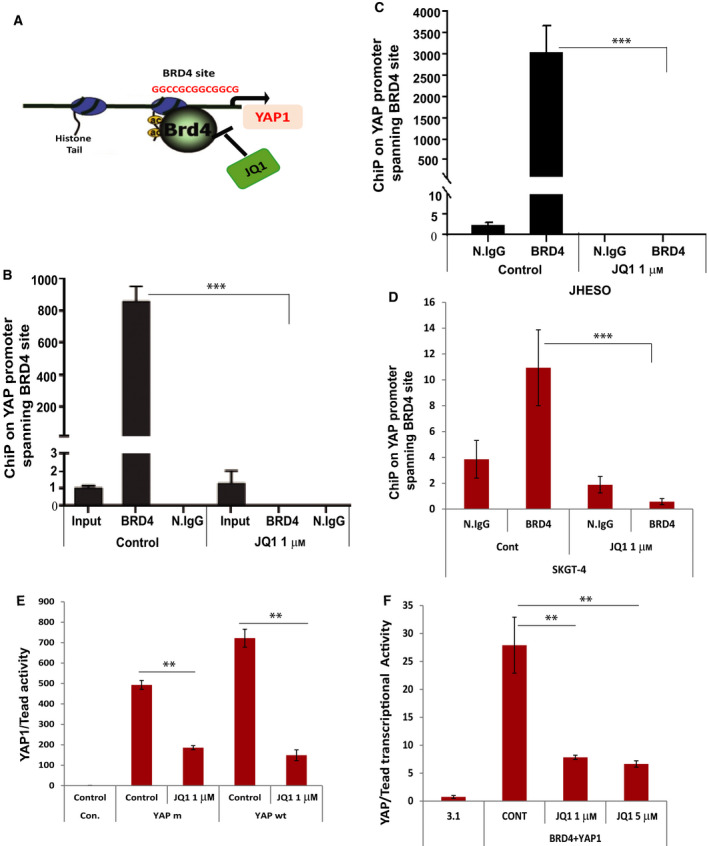
Bromodomain‐containing protein 4 regulates YAP1 transcription through binding its promoter and JQ1 suppresses BRD4‐mediated YAP1 transcription. (A) A diagram depicting that BRD4 regulates YAP1 through binding its proximal promoter at the BRD4 site, while JQ1 blocks the BRD4 regulation of YAP1. (B) Quantitative ChIP assay was performed using primers spanning the BRD4 binding site in the *YAP1* promoter in chromatin pulled down by BRD4 antibody or normal IgG in 293T cells treated with or without JQ1 at 1μm for 48 h**.** (C and D) Quantitative ChIP assays were performed using primers spanning the BRD4 binding site in the *YAP1* promoter in chromatin pulled down by BRD4 antibody or normal IgG in JHESO and SKGT‐4 EAC cells treated with or without JQ1 at 1μm for 48 h. (E) YAP1/Tead transcriptional activity was determined by cotransfection of Gal4‐Tead and 5XUAS‐luciferase with either mutant or wt YAP1 cDNA with or without JQ1 treatment at 1μm for 48 h in JHESO cells. (F) Transcriptional activity of YAP1/Tead was detected by cotransfection of Gal4‐Tead and 5XUAS‐luciferase with YAP1 and BRD4 cDNA and then treated with JQ1 at 1** **μm and 5 μm for 48 h in JHESO cells. **P < 0.05; ***P < 0.01.

It is known that BRD4 together with its other homologue BRD2 and BRDT is acetylated chromatin readers to epigenetically regulate chromosome remodeling and facilitate gene transcription. It has been emerging that BRD4 enhances the transcription of key cell cycle genes through directly binding their promoters (Mochizuki *et al.*, 2008). When searching YAP1 promoter, we found a complete BRD4 binding motif (GGCCGCGGCGGCG) in YAP1 upstream of the transcriptional start site as indicated in Fig. 3A. To determine whether BRD4 is recruited to the promoter of YAP1 and whether JQ1 can block the binding of BRD4 in the promoter of YAP1 in EAC cell lines, we performed chromatin immunoprecipitation assays in HET293T and SKGT‐4 and JHESO EAC cells transfected with BRD4 and treated with JQ1 (Fig. 3B–D). We performed quantitative polymerase chain reaction analysis using a pair of YAP1 promoter primers spanning the BRD4 binding site (Fig. 3A). Immunoprecipitation of BRD4‐associated chromatin selectively enriched DNA fragments of the YAP1 promoter containing the BRD4 binding site, whereas treatment with JQ1 markedly suppressed BRD4 binding to the YAP1 promoter in HET293T (Fig. 3B) and two EAC cell lines (Fig 3C,D). Consistently, JQ1 significantly reduced YAP/Tead transcriptional activity induced by either mutant or wild‐type YAP1 (Fig. 3E) and decreased YAP1/TEAD transcriptional activity induced by YAP1 and BRD4 cotransfection in JHESO cells and Flo‐1 cells (Fig. 3F). These data supported that BRD4 as a coactivator enhances YAP1 transcription and downstream signaling in EAC cells and that treatment with JQ1 suppresses BRD4‐mediated YAP1 expression and its transcriptional output.

### 3.4 JQ1 preferentially inhibits YAP1 in EAC cells and suppresses YAP1‐mediated tumorigenesis *in vivo*

**Fig. 4:**
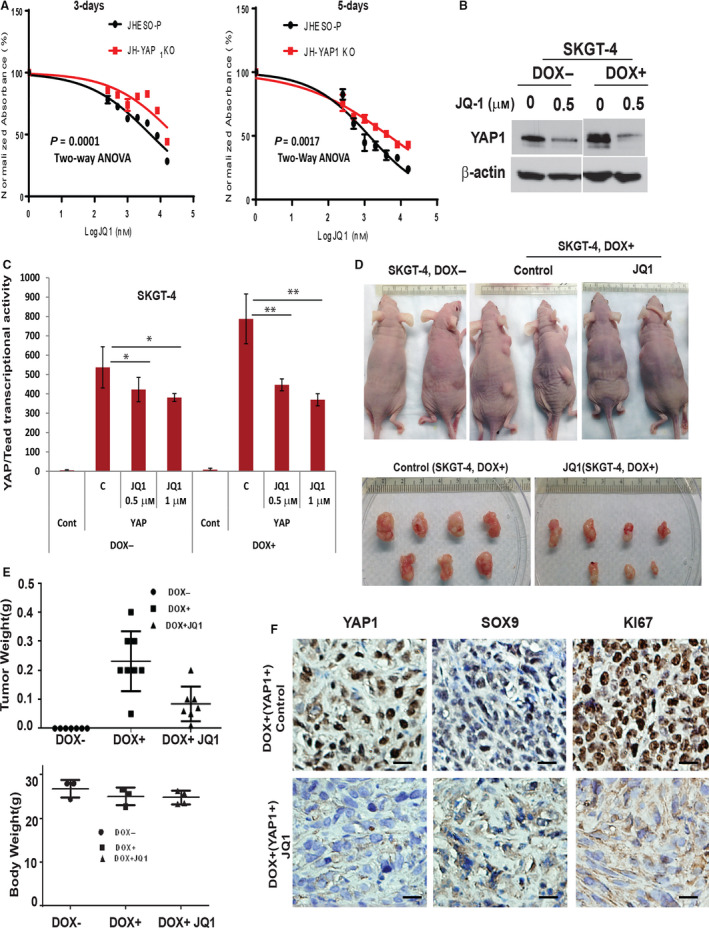
JQ1 preferentially inhibits YAP1‐mediated tumorigenesis *in vivo.* (A) JHESO parental cells with YAP1 high and JHESO cells with YAP1 knockout were treated at various concentrations (0, 0.25 μm, 0.5 μm, 1 μm, 2 μm, 4 μm, 8 μm, and 16 μm) of JQ1 for 3 days and 6 days, and then, cell growth was measured at absorbance reading at OD 490 (*P* = 0.0001 and *P* = 0.0017, respectively). (B) Expression of YAP1 was determined by immunoblotting in SKGT‐4 with YAP1 induction (DOX+) or without (DOX‐) treated with JQ1 at indicated dosage for 48 h. (C) YAP1/Tead luciferase activities were revealed by cotransfection of Gal4‐Tead, 5XUAS‐luciferase, and YAP1 cDNA plasmids and Renilla into SKGT‐4 (PIN20YAP1) cells and then treated with JQ1 at 0.5 μm and 1 μm. After 48 h of transfection, luciferase reporter activities were measured according to Materials and Methods. Values shown represent the mean and SD of at least triplicate assays for all experiments. (D) SKGT‐4 (PIN20YAP1) cells with (DOX+) or without (DOX−) YAP1 induction were inoculated into nude mice of both sites (*n* = 5 per group). Representative tumors after 6 weeks are shown (D). Scale bar: 20 μm. After 6 weeks, tumor weight (E, top panel) and body weight (E, low panel) were measured as described in Materials and Methods. (F) Expression of YAP1, SOX9, and Ki67 was detected in mouse tumor tissues derived from SKGT‐4 (PIN20YAP1) xenograft nude mice using immunohistochemistry. 200x magnification. Scale bar: 25 μm.

To elucidate whether JQ1‐mediated inhibition of EAC cell growth depended on YAP1 levels, we first treated JHESO cells with YAP1 high and YAP1 knockout and found that knockout of YAP1 in JHESO cells significantly decreased the sensitivity to JQ1 treatment at 3 days and 6 days (Fig. 4A), indicating JQ1 inhibition of EAC cell growth depended on YAP1 levels (Fig. 4A). To further determine whether JQ1 preferentially suppresses YAP1 expression and its transcription, we utilized our established lentiviral‐inducible SKGT‐4 cells (pIN20YAP1) with (DOX+) or without (DOX‐) YAP1 induction. As shown in Fig. 4B, JQ1 at relatively low concentration (0.5 μm) preferentially inhibited expression of YAP1 in SKGT‐4 DOX+ cells in comparison with SKGT‐4 DOX‐ cells (Fig. 4B). YAP1/Tead1 transcriptional activity in SKGT‐4 DOX+ cells was significantly more reduced than that in SKGT‐4 DOX‐ cells upon treated with JQ1 at 0.5 μm and 1 μm for 24 h (Fig. 4C). *In vivo*, we implanted both SKGT‐4 DOX‐ and DOX+ into nude mice and found that only SKGT‐4 DOX+ cells were able to form tumors (Fig. 4D). When we applied JQ1 at 15mg kg^−1^ three times a week in mice bearing SKGT‐4 DOX+ tumors, we found that JQ1 dramatically decreased tumor growth in mice bearing SKGT‐4 DOX+ tumors (Fig. 4D,E) without apparently affecting mice body weights (Fig. 4E, lower panel). When further analyzing tumors treated with JQ1, we found that the level of proliferation markers Ki67, YAP1, and SOX9 was greatly reduced by treatment of JQ1 (Fig. 4F). These data indicate that JQ1 could be a novel class of YAP1 inhibitor that preferentially suppresses YAP1 expression and YAP1‐induced oncogenesis in EAC cells.

### 3.5 JQ1 selectively inhibit CSC population and tumor cell growth in resistant EAC cells

**Fig. 5:**
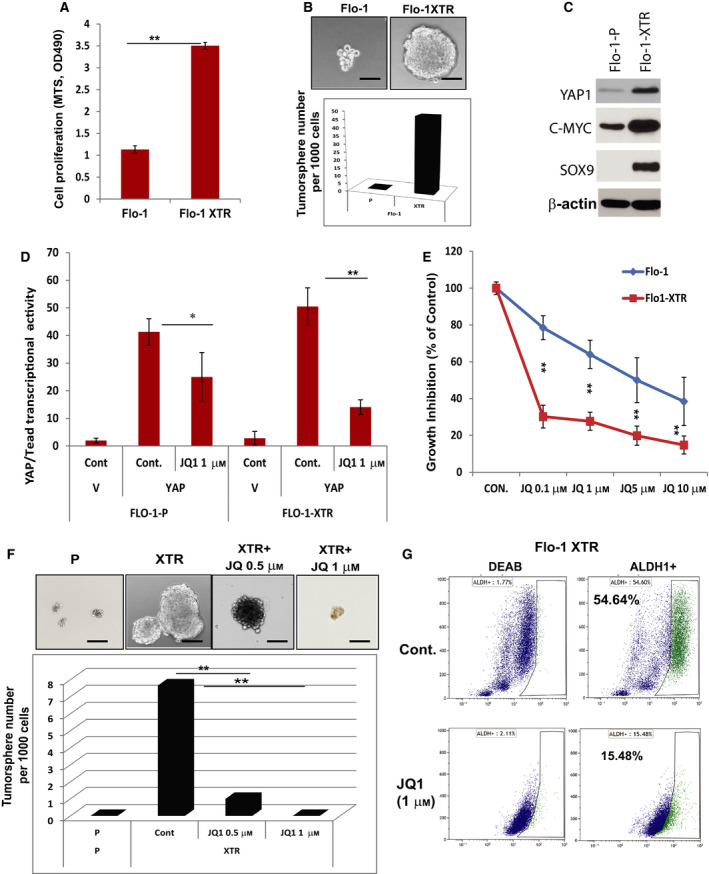
The JQ1 selectively inhibits CSC population and tumor cell growth in therapy‐resistant EAC cells. (A) The survival of Flo‐1 and its resistant Flo‐1 XTR cells was determined using MTS assay at day 6 day as described in Materials and Methods. (B) Representative image (top) and quantification (low) of tumorsphere formation in Flo‐1 and Flo‐1 XTR cells were demonstrated. Scale bar, 50 μm; (C) Expression of YAP1, c‐MYC, and SOX9 was determined by immunoblotting in Flo‐1 parental and radiation‐resistant cells (XTR). (D) YAP/Tead transcriptional activity was determined by cotransfection of Gal4‐Tead and 5XUAS‐luciferase and YAP1 cDNA in Flo‐1‐P and Flo‐1 XTR cells treated with JQ1 at 1 μm. (E) Effects of JQ1 on both Flo‐1‐P and Flo‐1 XTR cells were determined using MTS assay as described in Materials and Methods. (F) Representative images (top) and quantification (low) of tumorsphere formation in Flo‐1, Flo‐1 XTR, and XTR cells treated with JQ1 at 0.5 μm and 1 μm for 10 days, and tumorsphere number will be determined under microscope. Experiments were repeated three times. Scale bar, 50 μm; (G) The proportion of ALDH1+ population in Flo‐1 XTR and Flo‐1 XTR cells treated with JQ1 was labeled using ALDH1 labeling kit (ALDEFLUOR^TM^ Kit, Stemcell Technology) according to the protocol

We have demonstrated that radiation‐resistant cells (Flo‐1 XTR) have increased cell proliferation and tumorsphere formation capacity accompanied by increased YAP1, SOX9, and c‐MYC expression (Fig. 5A–C). We found that JQ1 selectively reduced YAP1/Tead transcriptional activity in Flo‐1 XTR cells compared with Flo‐1 parental cells (Fig. 5D). We observed that JQ1 preferentially inhibited Flo‐1 XTR cell growth compared with parental Flo‐1 cells (Fig. 5E), which were accompanied by greatly reduced YAP1/Tead transcriptional activity in Flo‐1 XTR cells (Fig. 5D). Furthermore, JQ1 dramatically reduced tumorsphere formation in Flo‐1 XTR cells, while Flo‐1 parental cells do not form tumorspheres (Fig. 5F). We have previously demonstrated that Flo‐1 XTR cells enrich CSC population compared with Flo‐1 parental cells labeled by ALDH1 labeling using ALDEFLUOR detection kit (Stemcell Technologies, Vancouver, BC, Canada) (Wadhwa *et al.*, 2017), while JQ1 suppressed the CSC population (ALDH1+) more than threefold in Flo‐1 XTR cells at 1 μm for 48 h (Fig. 5G). These data suggested that radiation‐resistant EAC cells enriched CSC properties and growth advantage could be blocked by JQ1, which is largely due to inhibition of YAP1 and its transcriptional activity.

### 3.6 Strong antitumor activity by JQ1 in combination with docetaxel *in vitro* and *in vivo*

**Fig. 6:**
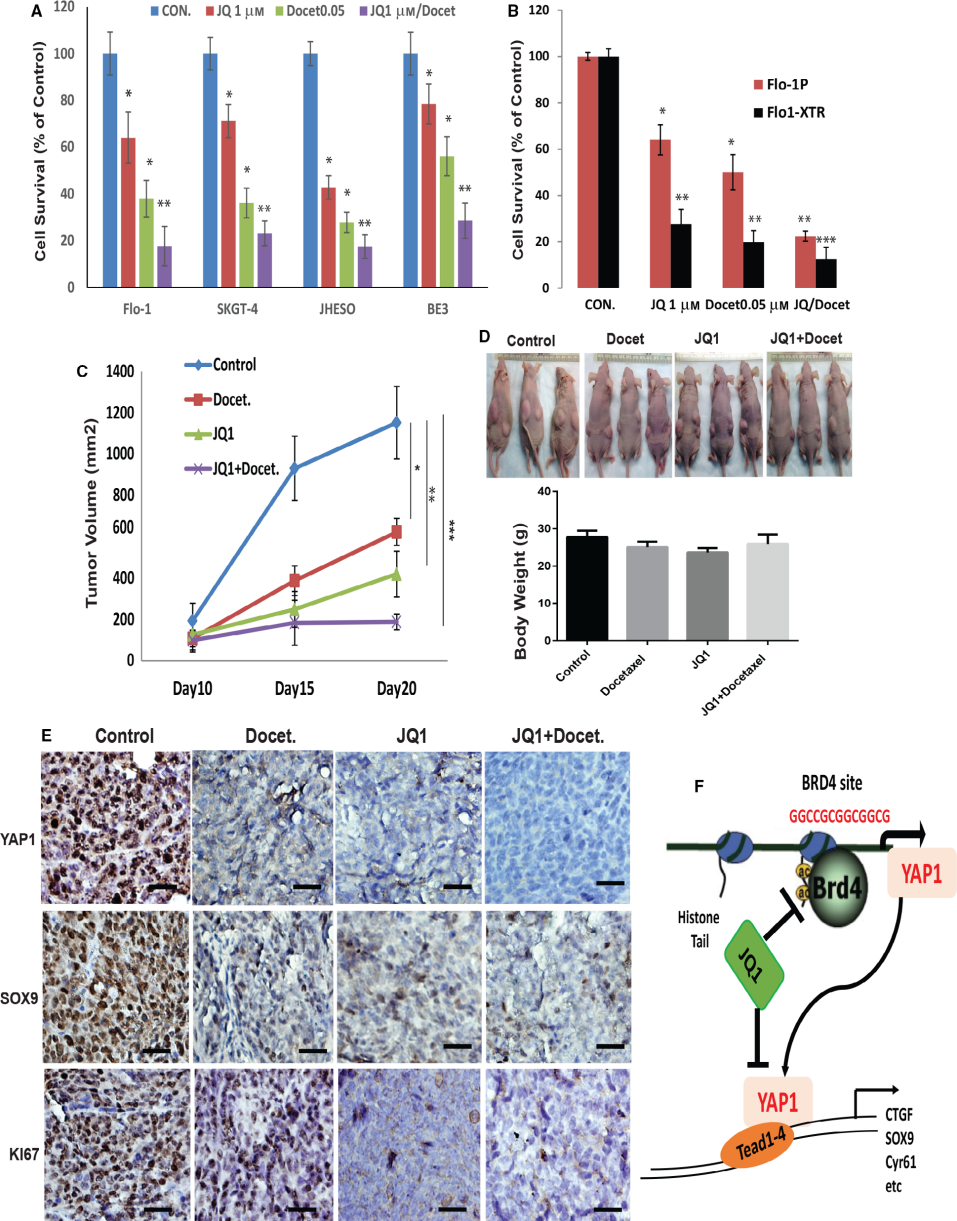
Strong antitumor activity by JQ1 in combination with docetaxel *in vitro* and *in vivo.* (A). Flo‐1, SKGT‐4, JHESO, and BE3 four EAC cell lines were treated with JQ1 and docetaxel either alone or in combination for six days at indicated dosage, cell survival was determined using MTS assay. *P < 0.05; and **P < 0.01. (B) Flo‐1 parental and its radiation‐resistant XTR cells (Flo‐1 XTR) were treated with JQ1 or docetaxel or their combination for six days at the concentration indicated, cell survival was determined using MTS assay. *P < 0.05; **P < 0.01;***P < 0.001. (C and D) JHESO cells (1.5 × 10^6^) were subcutaneously injected in nude mice on both sites (left, right) injections; Mice were treated with either JQ1 alone, docetaxel alone, or in combination as described in Materials & Methods. 5 mice/group. Mouse body weight and tumor volume in each group were measured and calculated as described in Materials & Methods. *P < 0.05; **P < 0.01; and ***P < 0.001. (E) Expression of Ki67, YAP1, and SOX9 was detected using immunohistochemistry in mouse tumor tissues derived from JHESO xenograft nude mice. Scale bar, 20 μm; 200x magnification. (F) Working model by which BRD4 regulates YAP1 and its transcriptional output, while JQ1 strongly blocks BRD4‐mediated YAP1 expression and its downstream targets

To further elucidate the therapeutic role for BET in YAP1‐driven tumors and find the right novel regimen for EAC therapy, we assessed the JQ1 in combination with docetaxel, the commonly used chemotherapy agent in the clinics *in vitro* and *in vivo*. First, we found that JQ1 decreased cell growth in four EAC cell lines including Flo‐1, SKGT‐4, JHESO, and BE3 EAC cells with high YAP1 and this became more effective when JQ1 was combined with docetaxel (Fig. 6A). More importantly, JQ1 preferentially suppressed radiation‐resistant Flo‐1 XTR cells compared with Flo‐1 parental cells and in combination with docetaxel demonstrated best antiproliferative effects (Fig. 6B). To investigate the antitumor efficacy of treatment with JQ1 combining with docetaxel *in vivo*, we tested JQ1 alone and/or in combination with docetaxel in the JHESO xenograft mouse model. We subcutaneously injected JHESO cells with high level of YAP1 into nude mice. We then randomly divided the mice bearing tumors into four groups as follows: control alone, JQ1 alone (30 mg per kg body weight intraperitoneally, three times a week), docetaxel alone (1 mg per kg body weight intraperitoneally, one time a week), or JQ1 and docetaxel. Tumor growth and tumor volumes were monitored and measured over 3 weeks of treatment. Xenograft tumor weights and mouse body weights were measured at the end of treatment. The results shown in Fig. 6C demonstrated JQ1 significantly decreased tumor growth in JHESO xenograft tumors, the combination treatment of JQ1 and docetaxel markedly reduced tumor volumes and showed the best antitumor activity, while body weights of mice among the groups did not differ significantly (Fig. 6D, lower panel). Furthermore, the expression of KI67, YAP1, and its target SOX9 in treated mouse tumors was significantly reduced upon treatment with the combination of JQ1 and docetaxel (Fig. 6E). Thus, treatment with JQ1 in combination with docetaxel demonstrated strong antitumor effects in EAC cells *in vivo*.

## 4 Discussion

We show, for the first time, that BRD4 is a critical regulator of YAP1 transcription and expression through direct occupancy of its promoter, while BET inhibition effectively decreased YAP1 expression and its transcription output (CTGF, SOX9, and Cyr61) in EAC cells (Fig. 6F). More interestingly, JQ1 can effectively suppress CSC properties and decrease ALDH1+ cells in radiation‐resistant EAC cells and this effect is further amplified by the addition of docetaxel *in vitro* and *in vivo*. Our data indicate that targeting nuclear oncogenic YAP1 can be achieved through BET inhibition in EAC and BET inhibition provides a new therapeutic strategy for EAC with high YAP1 and therapy‐resistant tumors (Fig. 6F).

Hippo/YAP1 has emerged as an important target in various cancer types including EC and often a terminal for many oncogenic pathways (Keren‐Paz *et al.*, 2015). However, how to target YAP1 in clinical setting is challenging. YAP1 antisense oligo or small‐molecule inhibitors are being developed in oncology space, but none is yet established. Verteporfin (VP) was first identified as a small‐molecule inhibitor of YAP1/TEAD association and suppressed YAP1’s oncogenic activity (Liu‐Chittenden *et al.*, 2012). Several reports have demonstrated the potential for VP in suppressing tumor growth and also serving as a research tool to study Hippo/YAP1 functions (Gibault *et al.*, 2017; Nguyen *et al.*, 2015). However, the utility of VP on cancer treatment is limited due to its toxicity. We have recently reported a novel YAP1 inhibitor CA3, which strongly suppressed YAP1 expression and YAP1 activity (Song *et al.*, 2018). Its clinical utility is under further investigation. C19 compound has been reported to inhibit YAP1/Tead luciferase reporter activity and showed antitumor activity through GSK3‐β‐mediated degradation of TAZ (an analog of YAP1) (Basu *et al.*, 2014). C19 is also a potential inhibitor for TGF‐β and Wnt signaling in addition to Hippo/YAP1/TAZ. Thus, effective specific YAP1 inhibition especially in EAC is lacking.

Bromodomain‐containing protein 4 is one of the major BET family members that interacts with chromatin and recruits other transcriptional factors to regulate gene expression (Belkina and Denis, 2012; Wu and Chiang, 2007). Increasing evidence suggests that BRD4 regulates many important genes and plays a critical role in tumor initiation, progression, and CSC maintenance. BRD4 regulates MYC to promote tumor growth in many tumor types (Ba *et al.*, 2018), and BET inhibition by JQ1 and its analog (FT1101, CPI‐0610, etc.) has shown activity in clinical trials (NCT02543879, NCT02158858, etc). It has been reported that BRD4 cooperates with twist to mediate twist‐dependent transcription programs in breast cancer tumorigenesis (Shi *et al.*, 2015). Recent studies from several groups have identified that BRD4 regulates cancer cell dissemination through upregulation of stem cell signaling, Jagged1/Notch and ALDH1A activity, while BET inhibition suppressed Jagged1/Notch signaling and ALDH1 activity by targeting Jagged1 promoter or ALDH1A1 superenhancer in breast or ovarian cancers (Andrieu *et al.*, 2016; Yokoyama *et al.*, 2016). BRD4 increases transcription of critical genes that are involved in embryonic stem cell maintenance and oncogenesis by regulating chromosome remodeling or directly binding to enhancers or promoters of target genes (Dey *et al.*, 2009; Loven *et al.*, 2013; Wu *et al.*, 2013). Recent study from Zanconato *et al.* (2018) demonstrated that YAP1/TAZ recruited BRD4 to chromatin and physically interacted with BRD4 to mediate their downstream targets.

However, the unique finding of this study is that BRD4 is a critical regulator for YAP1 transcription and expression. Targeting YAP1 can be achieved by BET inhibition. We found that transfection of BRD4 in EC cells significantly upregulated YAP1 expression, nuclear localization, and its downstream output through directly binding YAP1 promoter, while BET inhibitor JQ1 effectively blocked BRD4 upregulation of YAP1. However, Tang Y et al revealed that BRD4 directly occupies Gli1 and Gli2 promoters, regulates Hh signaling, and promotes Hh‐driven tumors, which can be inhibited by JQ1 (2014). A recent report by Zhang *et al *(2017) found that JQ1 negatively regulates c‐MYC, Bcl‐XL, and YAP1 and induction of p21 and p27; however, how JQ1 suppresses YAP1 is not known. When searching the YAP1 promoter, we found a complete BRD4 binding motif (GGCCGCGGCGGCG) (Hussong *et al.*, 2014) in YAP1 upstream of the transcriptional start site. Our chromatin immunoprecipitation (ChIP) assays confirmed that immunoprecipitation of BRD4‐associated chromatin selectively enriched DNA fragments of the YAP1 promoter containing the BRD4 binding site, whereas JQ1 significantly suppressed BRD4 binding to the YAP1 promoter in several EAC cell lines identified by ChiP assay. The mechanisms of BRD4 regulation of YAP1 proposed in our study is significantly different from that proposed in the study of Zanconato F et al (2018). Our study indicates that BRD4 as a coactivator of YAP1 enhances YAP1 transcription and downstream signaling, whereas JQ1 effectively suppresses BRD4‐mediated YAP1 transcription and expression in EAC cells.

## 5 Conclusions

Our study is clinically relevant and important for EAC patients with YAP1 high expression and/or those with therapy resistance. We found that BRD4 is a critical regulator for YAP1 transcription and expression. Targeting YAP1 can be achieved by BET inhibition. Our data indicated that resistant tumors are enriched in CSCs and YAP1/BET inhibition by JQ1 significantly suppresses resistant tumor cell growth, tumorsphere formation, and reduced population of CSCs. Most importantly, YAP1/BET inhibition significantly enhances antitumor effects of conventional cytotoxic (docetaxel) when combined *in vitro* and *in vivo*. Thus, this study provides a new regimen for a novel potential clinical trial on EAC patients when they fail after their first‐line treatment (chemo/radiation) in the clinic. Large preclinical studies using novel BET inhibitors and using human patient‐derived tumor cells are needed and warranted before clinical trial that is currently under our active investigation.

## Author contributions

S Song conceived and designed the study. SS, YL, YX, JKJ, and LM developed methodology. SS, YL, YX, JKJ, LM, AWS, WZ, XD, BL, and MPP contributed to the acquisition of data. SS, YL, YX, JAA contributed to analysis and interpretation of data (statistical and bioinformatics, etc.). SS, YL, AS, LFH, JKJ, LM, RLJ and JAA wrote, reviewed, and revised the manuscript. SS and JAA provided administrative, supervision, and materials/financial supports.

## Conflict of interest

The authors declare no conflict of interest.

## Consent to participate and ethics approval

The human subject protocol and animal protocol for this study were approved by the Institutional Review Board of UT MD Anderson Cancer Center.

## Consent for publication

All authors approved to publish the study in this journal.

## Availability of supporting data

The materials used and datasets used and/or analyzed in this study are available from the corresponding author on reasonable request.

## Supporting information


**Fig. S1.** Anti‐proliferation effects of JQ1 on five EAC cell lines.
**Fig. S2.** BRD4 was associated with tumor size and poor survival in EAC patients.
**Fig. S3.** BRD4 increased YAP1 and JQ1 suppressed YAP/TEAD transcriptional activity mediated by either WT or mutant YAP1 at Ser127.
