# Supplementary material for: Targeting Hippo coactivator YAP1 through BET bromodomain inhibition in esophageal adenocarcinoma
Source: Mol Oncol. 2020 Apr 7;14(6):1410–26. doi: 10.1002/1878-0261.12667 (PMC7266288; doi:10.1002/1878-0261.12667)
Supplement: Supplementary file 1 — Fig. S1. Anti‐proliferation effects of JQ1 on five EAC cell lines. Fig. S2. BRD4 was associated with tumor size and poor survival in EAC patients. Fig. S3. BRD4 increased YAP1 and JQ1 suppressed YAP/TEAD transcriptional activity mediated by either WT or mutant YAP1 at Ser127. [file MOL2-14-1410-s001.pdf]

Supplemental Figure legends

Supplemental Figure 1. Anti-proliferation effects of JQ1 on five EAC cell lines. The cells were treated with JQ1 at concentration of 0.25μM, 0.5 μM, 1 μM, 2 μM, 4 μM, 8 μM and 16 μM for 3 day and 6 day treatment and then CellTiter96 Aqueous one solution was added to each well (20ul to 100ul culture media) followed by 2 hours incubation at 37 °C and absorbance reading at OD 490. GraphPad Prism7 was used for cell proliferation inhibition analysis by following the instruction from GraphPad.com (https://www.graphpad.com/support/faq/how-to-determine-an-icsub50sub/).

Supplemental Figure 2. BRD4 was associated with tumor size and poor survival in EAC patients

A. 87 cases of EAC tumor tissues were detected with BRD4 mRNA level using Q-PCR and Patients were divided into two groups by tumor size, >5cm group or >=5cm and <= 5cm group or <5cm. Unpaired t test was used to compare the mRNA relative level of BRD4 between the two groups. *P <0.001; **B**. The association of BRD4 and patients' survival were analyzed from TCGA data set in two independent patients' cohorts (http://Kmplot.com/analysis).

Supplemental Figure 3. BRD4 increased YAP1 and JQ1 suppressed YAP/TEAD transcriptional activity mediated by either WT or mutant YAP1 at Ser127. A. Expression of BRD4 cDNA significantly increased YAP1 and its targets-SOX9 and CTGF mRNA level detected by quantitative-PCR in KATOIII cells. **P<0.01. **B**. Transfection of BRD4 cDNA increased YAP1 and SOX9 expression in both JHESO and Flo-1 cells determined using Western blotting. **C**. CTGF mRNA level was detected by quantitative-PCR in JHESO cells.*p<0.05; **P<0.01. **D**. YAP1/Tead transcriptional activity was determined by co-transfection of Gal4-Tead and 5XUAS-luciferase, wide type YAP1 cDNA or YAP1 mutant cDNA (CMV-S127A-YAP) into SKGT4 cells and then treated with JQ1 at 1mM for 48 hours as described as Materials&Methods. For all experiments, values shown represent the mean and SD of at least triplicate assays *P< 0.05; **P<0.001.

Supplemental Figure 1.

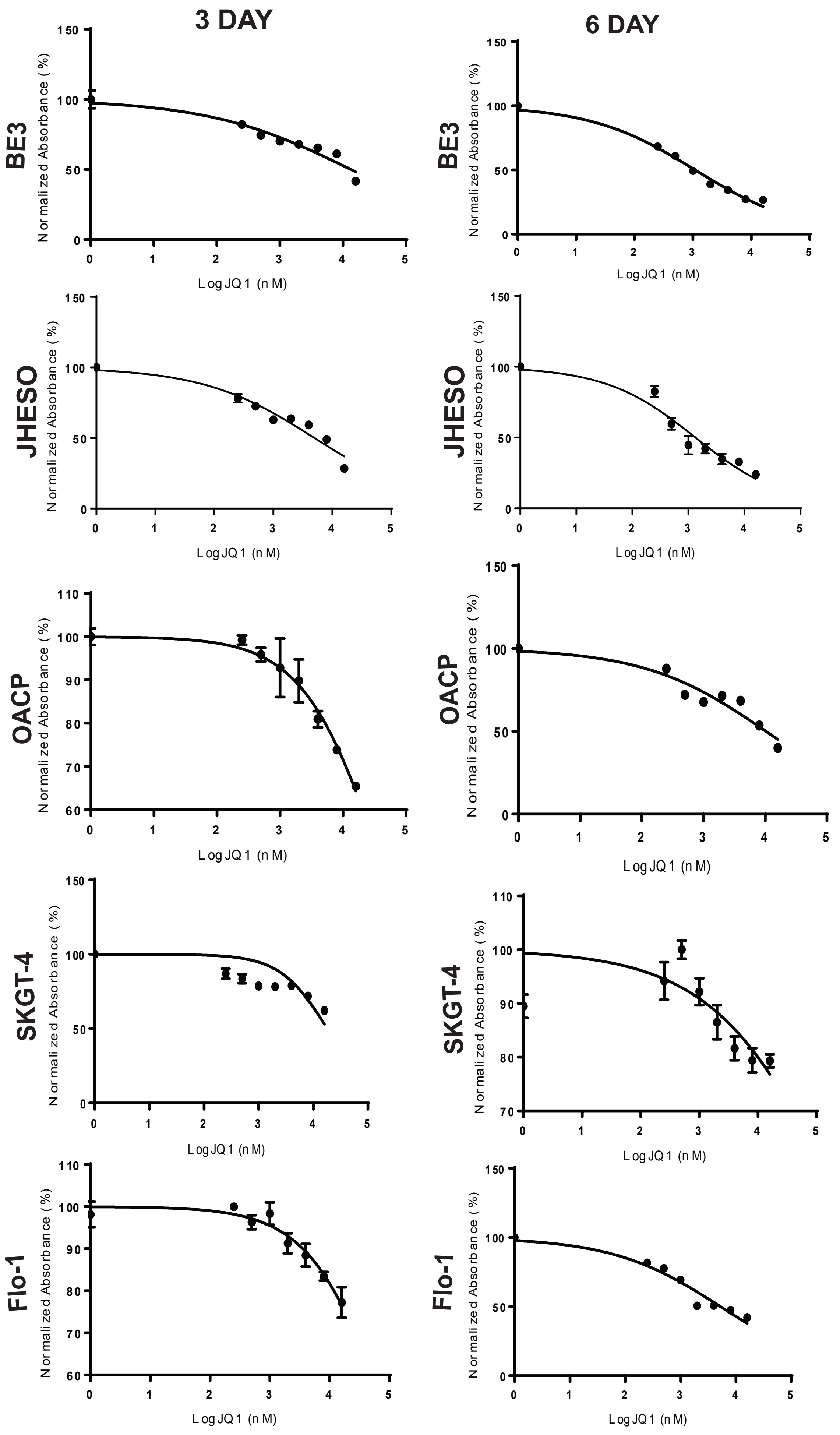

Supplemental Figure 2.

A.

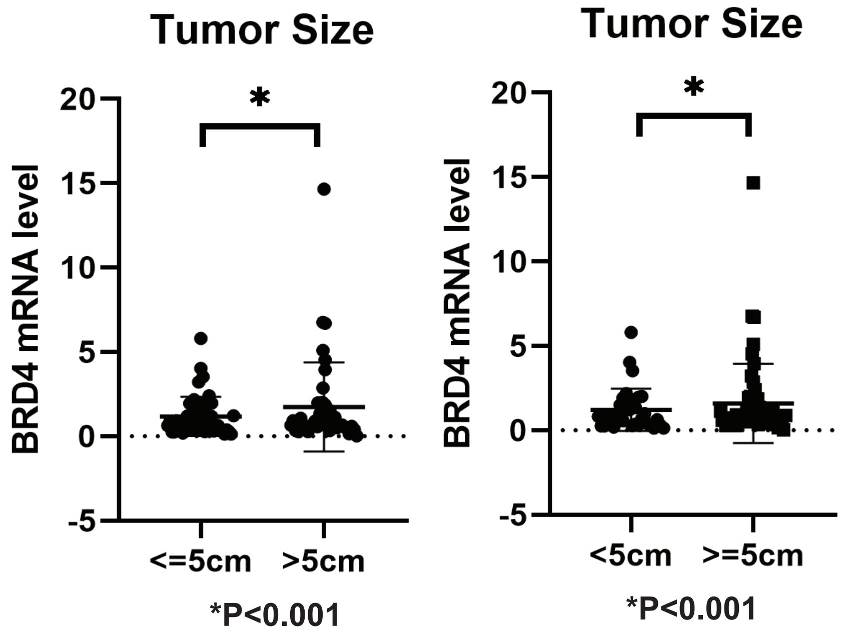

B.

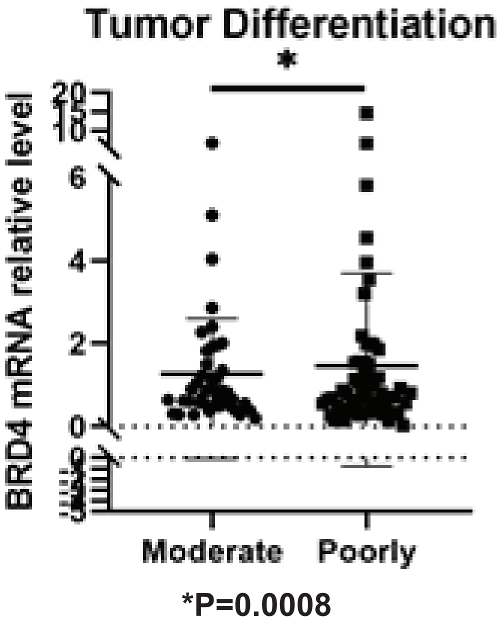

C.

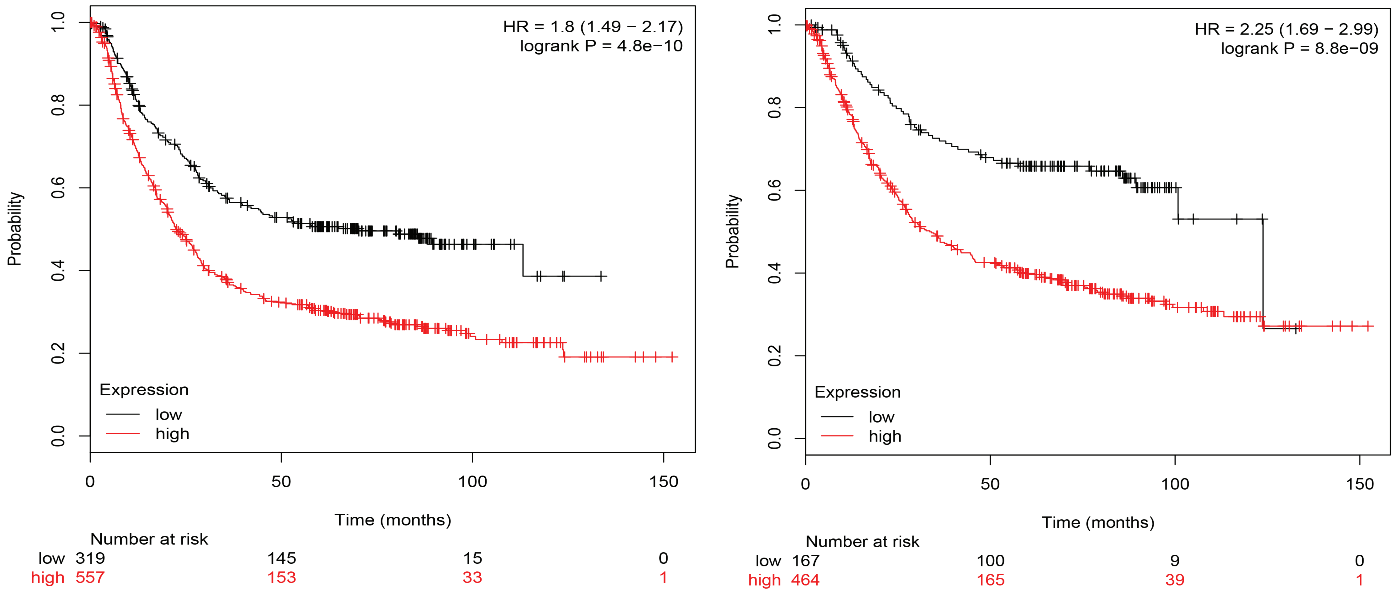

P <0.001

Suppplemental Figure 3.

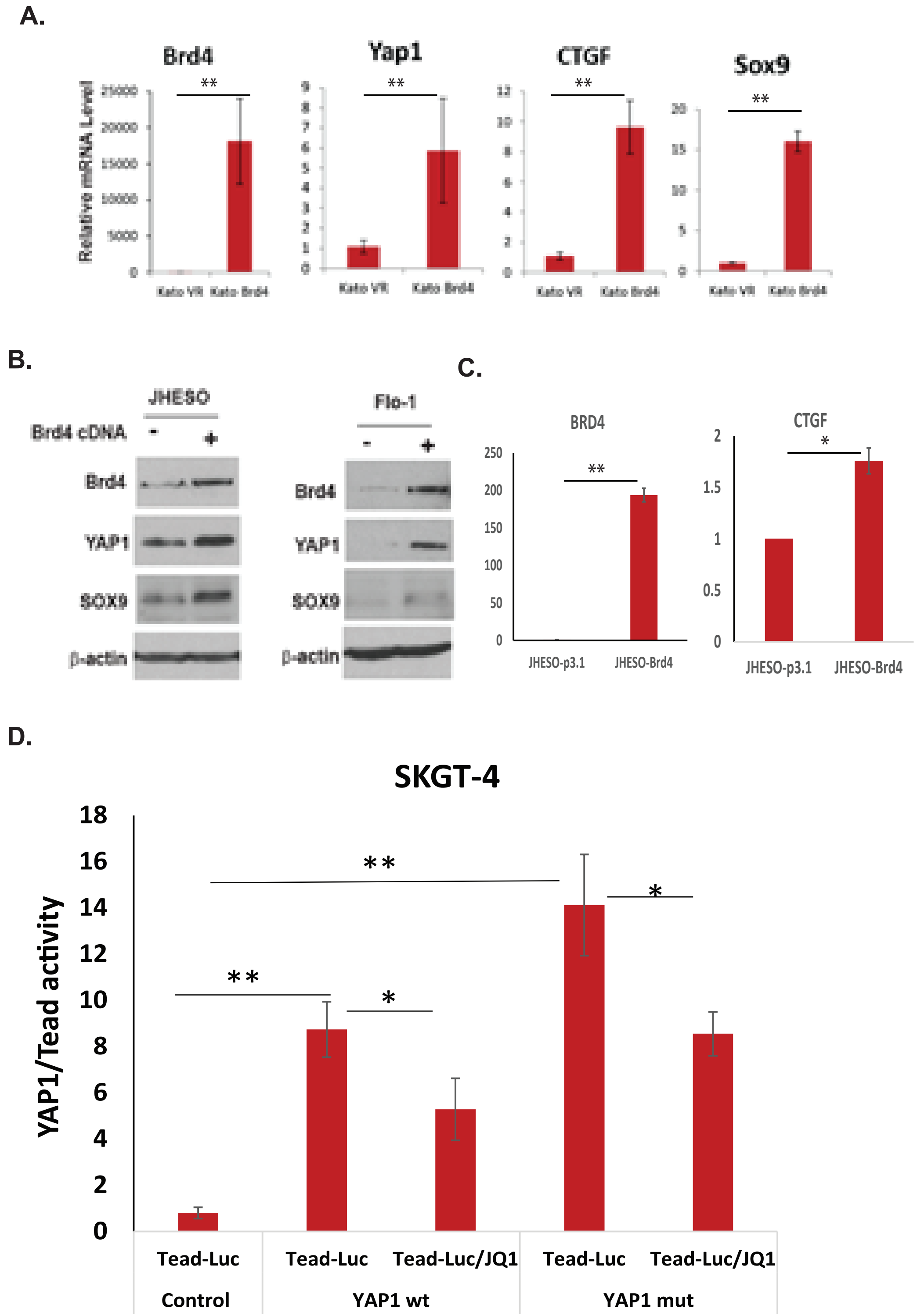

*P<0.05; **P<0.001
